# Insights into Antioxidant Activity and Trace Element Distribution of Aqueous Extract of *Silybum marianum* Seeds

**DOI:** 10.3390/molecules31061034

**Published:** 2026-03-19

**Authors:** Li Quan, Yi-Xiao Wang, Xiu-Lan Cai, En-Chao Zhou, Xue-Wen Guo, Yi-Jun Chen, Hong-Zhen Lian

**Affiliations:** 1 Jiangsu University Key Laboratory of Tonifying Kidney and Anti-Senescence, The First Clinical Medical College, Nanjing University of Chinese Medicine, Nanjing 210023, China; 039317110@njucm.edu.cn; 2 State Key Laboratory of Analytical Chemistry for Life Science, School of Chemistry & Center of Materials Analysis, Nanjing University, Nanjing 210023, China; 602023240128@smail.nju.edu.cn (Y.-X.W.);

**Keywords:** *Silybum marianum*, aqueous extract, trace elements, antioxidant activity, RP-HPLC, ICP-MS, EPR

## Abstract

The purpose of this work is to investigate the binding state of inorganic elements to flavonoid components in aqueous extract of *Silybum marianum* (SM) seeds, as well as the antioxidant activity of the extract. This study employed reversed-phase high-performance liquid chromatography (RP-HPLC) to separate silymarin flavonoids in boiling water decoction of SM seeds, and collected the post-column effluent in the segments according to the retention time of seven main silymarin flavonoid components. Inductively coupled plasma mass spectrometry (ICP-MS) was subsequently utilized to quantify nine inorganic elements (As, Cd, Co, Cr, Cu, Fe, Mn, Mo, Zn) in the collected HPLC fractions of the decoction. Meanwhile, electron paramagnetic resonance spectroscopy (EPR) was employed to assess the free radical scavenging activity of aqueous extract of SM seeds, using the signal intensity changes of 2,2-diphenyl-1-picrylhydrazyl (DPPH) and DMPO-OH• adducts as quantitative metrics. The results showed that essential trace elements (Cu, Fe, Mn, Zn) mainly existed as inorganic ions or strong polar forms in the tea-like infusion, with weak binding to flavonoid compounds. On the other hand, the aqueous extract exhibited significant •OH scavenging capacity, with a scavenging rate of 95% against •OH generated by continuous 5 min ultraviolet irradiation of H_2_O_2_ aqueous solution. This study provides experimental evidence for the development of SM as a food–medicine dual-purpose resource, proposing that consumption of SM seed tea represents a facile and effective approach to supplement trace elements and intake silymarin for enhancing endogenous antioxidant defense.

## 1. Introduction

*Silybum marianum* (SM), commonly known as milk thistle, is an annual or biennial herb belonging to the Asteraceae family. Widely distributed globally, it has long been renowned for its hepatoprotective effects for centuries [1]. Modern pharmacological studies have indicated that SM exhibits significant antioxidant activity, with silymarin as its core bioactive component-a complex mixture primarily composed of flavonolignans. These include taxifolin (TXF), silycristin (SCN), silydianin (SDN), silybin A (SBNA), silybin B (SBNB), isosilybin A (ISBNA), and isosilybin B (ISBNB) [2,3]. In particular, the hepatoprotective and antioxidant effects of silymarin have been widely confirmed [4,5]. Studies have shown that silymarin can effectively reduce lipid oxidation and inhibit the formation of toxic oxidative products [6]. For instance, Akkaya and Yilmaz [7] indicated that SM extract significantly protects unsaturated fatty acids from free radical damage by reducing the level of lipid peroxidation, thereby exerting a potent inhibitory effect on lipid peroxidation. Further studies have revealed that various active components in SM extract also possess multiple pharmacological activities, such as hypoglycemic, immunomodulatory, antiviral, anti-inflammatory, and antitumor effects [8,9,10,11], thus exhibiting broad application prospects in the treatment of various related diseases. For example, a study has confirmed that silybin, an equimolar mixture of silybin A and silybin B, acts as an aldose reductase inhibitor, which can effectively reduce the risk of diabetes-related complications, including diabetic nephropathy, diabetic neuropathy, and steatohepatitis [11]. Flavonoids and other active substances extracted from SM seeds exhibited prominent performance in 2,2′-azino-bis(3-ethylbenzothiazoline-6-sulphonic acid) and 2,2-diphenyl-1-picrylhydrazyl (DPPH) radical scavenging assays as well as protein tyrosine phosphatase 1B inhibitory activity tests, by virtue of their excellent antioxidant activity and α-glucosidase inhibitory activity, thus holding potential as hypoglycemic agents [12]. In addition, the antioxidant and antitumor properties of silymarin also enable it to serve as a potential anticancer agent [13].

The biological activities of SM extracts are closely correlated with the extraction methods employed. Kim et al. [14] conducted in vitro antioxidant activity studies on aqueous extracts, ethanol extracts, and their active components derived from different parts of SM. The results demonstrated that the ethanol extracts of SM seeds contained significantly higher levels of silymarin and its components (taxifolin, silydianin, and silybin), thereby exhibiting more potent free radical scavenging capacity. Aldayel et al. [15] pretreated SM extracts with laser irradiation for 6 min and found that this method could increase the contents of silybin A and silybin B, which in turn enhanced the antibacterial activity of the extracts. Lekmine et al. [16] prepared the SM extracts using microwave-assisted extraction, which were verified to possess significant antioxidant, antidiabetic, and antibacterial activities. In addition, SM and natural silymarin can also be used as dietary supplements to enhance the antioxidant capacity of the organism [17], as well as food preservatives rich in antioxidant components [18]. Very interestingly, Clausen group indicated that hot water is a milder and greener solvent for the extraction of silymarin flavonoids from thistle seeds [19].

In the context of measuring and comparing antioxidant activity and identifying free radicals in biologically derived samples, electron paramagnetic resonance (EPR) spectroscopy integrated with spin-trapping techniques ranks among the most effective and insightful tools in modern analytical chemistry [20]. This technique is ideally suited for assessing the antioxidant properties of herbal extracts, given its ability to achieve direct, high-sensitivity detection without the confounding interference and side reactions inherent to spectrophotometric and fluorometric methods [21,22,23]. Notably, studies exploring SM extract antioxidant activity using EPR spectroscopy are surprisingly limited to date.

High-performance liquid chromatography (HPLC) is the most frequently used technique for silymarin flavonoid separation and analysis because of good selectivity, high efficiency, fast speed and excellent reproducibility [24]. Our research group developed a reversed-phase high-performance liquid chromatography (RP-HPLC) using methanol as an organic modifier of mobile phase with UV-Vis detection to separate and analyze seven common silymarin flavonoids from SM with boiling water, which was further validated by liquid chromatography–mass spectrometry (LC-MS). It was confirmed that the multiple decoctions at 100 °C like infusion of tea is a potential approach for uptake of the effective flavonoid components [25].

At present, the research on SM has mainly focused on the extraction technology and antioxidant activity of silymarin, whereas studies on the inorganic elements in SM remain relatively scarce [26]. In traditional Chinese medicine (TCM) theory, however, therapeutic efficiency of a natural herbal medicine is the integrated outcome of various effective components including secondary metabolites, metals and even the combined forms between them. It is reported that magnesium lithospermate B extracted from Salvia miltiorrhiza has Na^+^/K^+^-ATPase inhibitory activity and anti-ischemic neuroprotection effects [27]. Although there has been no research on the synergetic antioxidant activity of the protogenous complexes of essential trace elements and silymarin flavonoids existing in SM, some researchers have reported that the biological activity of artificially synthesized complexes of metal ions and flavonoid compounds is higher than that of using flavonoid compounds alone [28,29]. For example, Cu complexes can enhance the antioxidant and DNA-intercalating properties of kaempferol, luteolin, fisetin and apigenin [30] and increase the radical scavenging activity of naringenin [31], and Zn complex also can strengthen the antioxidant activity and cytotoxicity against cancer cells of rutin [32]. The analysis of trace elements provides a scientific basis for elucidating traditional pharmacology, toxicology, and the quality of Chinese medicinal materials, and offers guidance for the cultivation and modern comprehensive utilization of Chinese herbs [33]. Therefore, some studies have reported the interaction between flavonoids and transition metal ions, particularly Fe^3+^ and Cu^2+^. Spectroscopic and mechanistic analyses have demonstrated that metal coordination typically occurs through deprotonated phenolic groups, especially catechol-type moieties, and that complex stability increases with pH due to enhanced phenolate formation [34,35]. The ability of flavonoids to chelate iron has been recognized as an important component of their antioxidant mechanism, as metal binding can inhibit Fenton-type reactions and reduce reactive oxygen species formation [34,36]. However, most previous investigations have focused on synthesized pure flavonoids in defined chemical environments, rather than in complex herbal decoctions containing multiple coexisting components and trace inorganic elements. Consequently, limited information is available regarding the distribution behavior of trace elements in aqueous decoction systems and their potential association with flavonoid fractions under realistic preparation conditions. As a natural plant resource, SM is also rich in a variety of essential trace elements for the human body, such as Fe, Zn, Mn and Cu, which play important roles in regulating enzyme activity and maintaining redox balance in the body. In the early study of our research group, an analytical method based on inductively coupled plasma atomic emission spectrometry (ICP-AES) and inductively coupled plasma mass spectrometry (ICP-MS) was established for the analysis of multiple trace elements in the boiling water decoction of SM seeds [37]. It was found that most of the elements determined are soluble in water and extracted almost completely after four tea-like decoctions. ICP-MS boasts distinct technical advantages, including a low detection limit (down to the ppt level), a wide dynamic linear range, minimal interference effects, high analytical precision, rapid detection speed, simultaneous determination of multiple elements, and the capability to provide isotopic information. However, this technique, like ICP-AES, is a destructive elemental analysis method and cannot directly obtain information related to the speciation including the binding state of elements towards flavonoids which is crucial to elucidate the synergic biological effect of inorganic elements and silymarin flavonoids.

This study first adopted a stepwise combination of RP-HPLC and ICP-MS. The former was used to fractionate the boiling water decoction of SM seeds based on the retention characteristics of major silymarin components, and the latter was subsequently employed to quantify nine inorganic elements in the collected fractions of the decoction, thereby preliminarily elucidating the distribution of these elements and their potential interactions with the flavonoid components. Secondly, electron EPR spectroscopy was utilized to evaluate the DPPH radical scavenging capacity of boiling water extract of SM seeds. Additionally, this technique was utilized to investigate the scavenging potential of the extract against hydroxyl radicals (•OH) in an aqueous phase system, by monitoring the signal inhibition of DMPO-OH• adducts. As the most reactive and cytotoxic among reactive oxygen species, •OH can induce extensive damage to critical cellular components, including DNA, lipids, and proteins in living organisms [38]. Consequently, appropriate supplementation with exogenous antioxidants has been demonstrated to reduce the risk of oxidative stress-related diseases [39]. Collectively, this study aims to provide experimental evidence for the rational exploitation of SM as a food–medicine dual-purpose resource, by verifying that direct consumption of SM seed tea enables simultaneous intake of the antioxidant silymarin and supplementation of essential trace elements for human metabolic homeostasis.

## 2. Results and Discussions

### 2.1. Analysis of Trace Elements in Decoction of SM Seeds

#### 2.1.1. Separation Performance of Silymarin Flavonoids

The HPLC chromatogram of the boiling water decoction of SM seeds is presented in Figure S1. The peaks from 1 to 7 were identified to be orderly TXF, SCN, SDN, SBNA, SBNB, ISBNA, and ISBNB, which are clearly separated.

**Figure 1:**
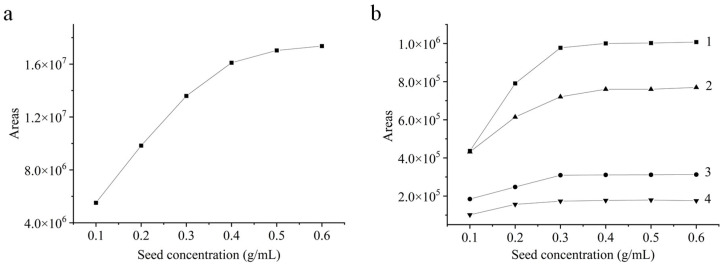
Relationship between peak area of silymarin flavonoids in boiling water decoction of SM seeds and seeds concentration in water: (**a**) TXF; (**b**) 1. SCN, 2. SBN, 3. SDN, 4. ISBN.

The separation performance of the silymarin flavonoids in boiling water decoction of SM seeds was evaluated at five different concentrations of seeds in water under the HPLC conditions. As illustrated in Figure 1, the extracted amounts of all the seven flavonoids in the boiling water tended to reach saturation when the concentration of SM seeds exceeded 0.4 g/mL. Once the seed concentration was higher than 0.2 g/mL, substantial amounts of oil were extracted therewith, which not only compromised the separation efficiency of most flavonoids but also increased the risk of column clogging. In contrast, when the seed concentration was below 0.1 g/mL, achieving the equivalent enrichment amounts should require a greater number of sample injections for HPLC separation, which prolonged the experimental duration, increased the total volume of collected eluents at lower concentrations, elevated the potential risk for sample contamination, and reduced the reproducibility of the experiment. Based on comprehensive comparison, the optimal concentration of SM seeds for subsequent experiments was adopted to be 0.1 g/mL.

#### 2.1.2. Element Concentrations in Fractionated Eluents

The blank solution was analyzed by ICP-MS under the conditions in Text S1 in 10 replicate measurements, and the limit of detection (LOD) for each element was defined as the concentration corresponding to three times the standard deviation (3 × SD). The LODs of As, Cd, Co, Cr, Cu, Fe, Mn, Mo, and Zn were determined to be 0.024, 0.005, 0.004, 0.020, 0.027, 2.400, 0.010, 0.030, and 0.200 ng/mL, respectively, indicating the high sensitivity of the established ICP-MS method. The quantification limits (LOQs) for these nine elements, defined as the concentration corresponding to 10 × SD, were 0.080, 0.017, 0.013, 0.067, 0.090, 8.00, 0.033, 0.100, and 0.667 ng/mL, respectively. Furthermore, a national certified reference material (CRM) of tea (GBW07605) was analyzed according to the same procedures. The results showed that the measured elemental contents (μg/g) of the tea CRM were in good agreement with the certified reference values (presented in parentheses)—that is, As: 0.25 (0.28 ± 0.04), Cd: 4500 (4300 ± 400), Co: 0.18 (0.18 ± 0.02), Cr: 0.84 (0.80 ± 0.03), Cu: 18.2 (17.3 ± 1.8), Fe: 263 (264 ± 15), Mn: 1282 (1240 ± 70); Mo: 0.043 (0.038 ± 0.007), and Zn: 26.6 (26.3 ± 2.0)—demonstrating the excellent accuracy and precision.

The results of element concentrations analyzed in the boiling water decoction of SM seeds are presented in Table 1, where the total concentration of each element in the digestion solution of effluents collected sequentially from the HPLC column was calculated as the sum of the concentrations across the nine digestions of fractions. It was found that the concentrations of essential trace elements Cu, Fe, Mn, and Zn (81.9, 63.6, 25.3, and 49.4 ng/mL, respectively) are relatively high among the nine elements determined. Moreover, the total amount (ng) of each element in the original decoction (50 μL × 4) was calculated by multiplying the total concentration (ng/mL) by the constant volume of 3 mL, and therefore a total 200 μL hot water decoction contained these four trace elements at 246, 191, 75.9, and 148 ng, respectively. Correspondingly, their total concentrations in the original 30 mL decoction obtained from 3 g of SM seeds reached 1230, 955, 380, and 740 ng/mL, respectively. These trace elements themselves possess certain biological activities or pharmacological effects, and they may directly participate in the overall efficacy of traditional Chinese medicine. Trace elements play a central role in the human antioxidant defense system. As key components of antioxidant enzymes, they effectively neutralize free radicals, reduce oxidative damage, thereby slowing aging and lowering the risk of chronic diseases.

The further analysis of the element concentrations in the fractions collected at different time intervals (Table 1) reveals that the overall concentrations of As, Cd, and Co are extremely low. This is presumably because these elements levels are low inherently, or they bind to some macromolecular substances in SM fruits, which are only marginally dissolved in boiling water. Notably, the concentrations of Cr, Cu, Fe, Mn, Mo, and Zn are the highest in the first fraction and far exceed those in all other following fractions. This observation is a reminder that these elements mainly exist in the form of free ions or in other highly polar species in the boiling water decoction, which exhibit very weak retention on the hydrophobic reversed-phase chromatographic column. The concentrations of Cr, Cu, Fe, Mn, and Zn are relatively high in the second and fourth fractions, implying that they may form weak interactions with certain polar compounds, e.g., small-molecule organic acids and TXF, in the decoction, resulting in partial retention on the chromatographic column. Moreover, the concentrations of Cr, Cu, Fe, Mn, Mo, and Zn do not vary obviously across all the subsequent fractions (5–9), which indicates that these elements do not bind to the major flavonoids in SM. Therefore, direct drinking of SM fruit tea could supplement some essential trace elements for the human body and maintain the balance of bodily metabolism. From another perspective, this finding also suggests that the extraction of SM flavonoids using organic solvents would lead to the loss of other beneficial trace elements and the disappearance of their synergistic effects.

### 2.2. Analysis of Antioxidant Activity of Aqueous Extract from SM Seeds

#### 2.2.1. DPPH Radical Scavenging Ability of the SM Extract

The findings and their implications should be discussed in the broadest context possible. Future research directions may also be highlighted. DPPH radicals possess a single-electron configuration, enabling their characteristic signals to be readily detected via EPR spectroscopy. In dilute solution, the unpaired electron interacts with two equivalent nitrogen nuclei (I = 1), resulting in a five-line hyperfine splitting pattern with an approximate intensity ratio of 1:2:3:2:1. As reported in our prior work [40], a distinct correlation has been established; the concentration dynamics of DPPH radicals can be quantitatively reflected by the double integral of the characteristic EPR spectra (encompassing all five hyperfine lines), which thus serves as a reliable metric for evaluating DPPH radical scavenging efficiency. The determination of DPPH radical SR relies on the redox reaction between DPPH radicals and free radical scavengers.

**Figure 2:**
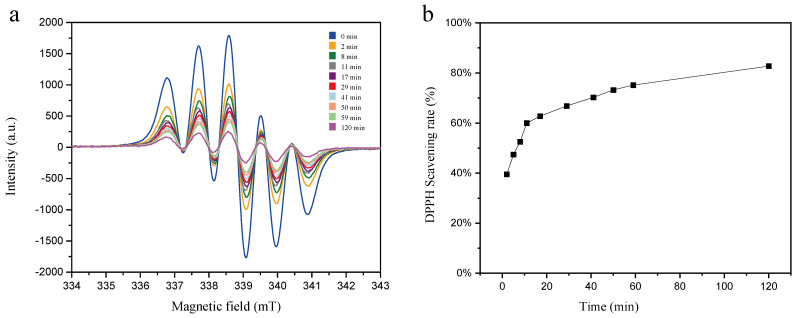
(**a**) EPR spectra of 0.5 mM DPPH solutions containing 0.5 mg/mL SM extract at different reaction time points; (**b**) time-dependent DPPH radical scavenging rate curve of 0.5 mg/mL SM extract against 0.5 mM DPPH radicals.

Figure 2 presents the time-dependent EPR spectra of 0.5 mM DPPH solutions supplemented with 0.5 mg/mL SM extract, as well as the corresponding DPPH radical scavenging activity profile of the extract over time. As depicted in the figure, the SR reached 39.5% merely 2 min after the initiation of the reaction. With prolonged reaction time, the SR increased progressively, attaining 75.2% at approximately 60 min. A further extension of the reaction duration to 120 min resulted in only a marginal increase in SR to 82.7%, indicating that the maximum scavenging capacity of the SM extract against 0.5 mM DPPH radicals is limited to this level.

**Figure 3:**
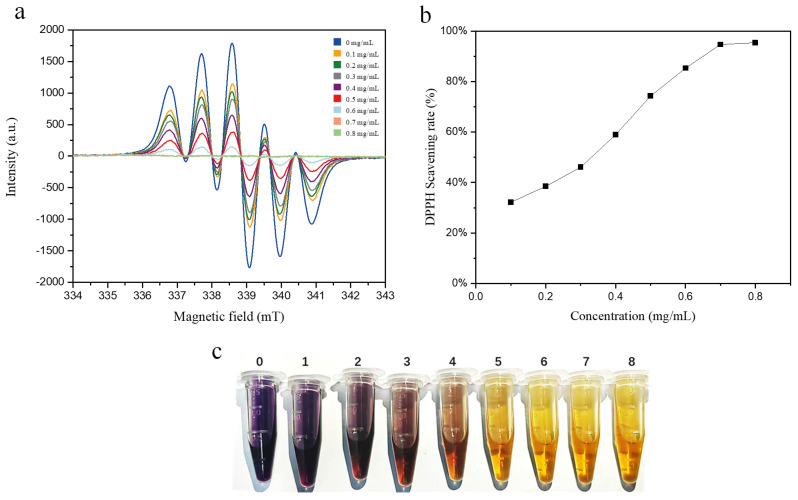
(**a**) EPR spectra of 0.5 mM DPPH solutions incubated with different concentrations of SM extract for 60 min. (**b**) DPPH radical scavenging rates of SM extract at different concentrations following 60 min incubation. (**c**) Visual appearance of 0.5 mM DPPH solutions treated with SM extract at gradient concentrations (0#–8#: 0.0, 0.1, 0.2, 0.3, 0.4, 0.5, 0.6, 0.7, and 0.8 mg/mL, respectively) after 60 min incubation.

The EPR signals and SR curves of 0.5 mM DPPH solutions following a 60 min reaction with SM extracts of varying concentrations are presented in Figure 3a,b. The results demonstrate that the DPPH scavenging rate of the 0.1 mg/mL SM extract reached 32.1%. As the extract concentration increased, the SR exhibited a linear upward trend, peaking at 94.7% at an SM extract concentration of 0.7 mg/mL. A further increase in extract concentration led to a plateau in SR at approximately 95.4%, suggesting that nearly all DPPH radicals in the system had been scavenged. In addition, the visual changes in solution color (Figure 3c) provide intuitive evidence of the radical-scavenging capacity of SM extracts. The blank DPPH solution displayed a characteristic purple color, whereas the solution color faded markedly upon the addition of SM extracts at relatively high concentrations. After 60 min of reaction, the solution turned pale yellow—especially when the SM extract concentration exceeded 0.6 mg/mL. By contrast, the DPPH solution treated with SM extracts at concentrations below 0.3 mg/mL retained its characteristic purple even after extending the reaction time to 120 min, which directly reflects a high residual content of unquenched DPPH radicals. Taken together, these results confirm that SM extracts at concentrations ranging from 0.1 to 0.8 mg/mL are capable of partially or completely scavenging 0.5 mM DPPH radicals.

#### 2.2.2. •OH Radical Scavenging Ability of the SM Extract

**Figure 4:**
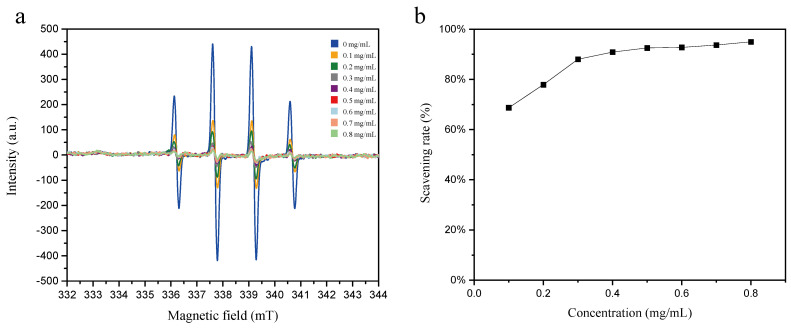
(**a**) Effect of SM extract concentrations (0.0–0.8 mg/mL) on EPR signal intensity of DMPO-OH• adducts (generated with 5 mM H_2_O_2_ + 1 mM DMPO). (**b**) The radical scavenging rates at different concentrations of SM extract.

The hydroxyl radical (•OH), an extremely reactive species, cannot be directly detected via EPR spectroscopy due to its high reactivity and short half-life. For its detection, •OH can be trapped by DMPO to form the relatively stable paramagnetic adduct DMPO-OH•. The detailed process for generating, capturing and detecting •OH is described as follows: A xenon lamp was employed to UV irradiate a mixture of H_2_O_2_ and DMPO. H_2_O_2_ decomposed to generate •OH, which was then subsequently trapped by DMPO to form DMPO-OH•. As illustrated in Figure 4, the EPR signal intensity and •OH radical SR of mixed solutions containing 5 mM H_2_O_2_, 1 mM DMPO, and different concentrations of SM extracts (0.1–0.8 mg/mL) after 5 min of UV irradiation revealed that the amplitude of the second characteristic peak in the EPR spectra decreased following the addition of SM extract in the system. With an increase in the concentration of SM extract in the system, the SR increased rapidly. Specifically, the SR of SM extract was 68.7% at a concentration of 0.1 mg/mL, reaching 92.5% at 0.5 mg/mL. Furthermore, the increment in •OH SR slowed as the SM concentration increased, ultimately attaining 95.0% at 0.8 mg/mL.

**Figure 5:**
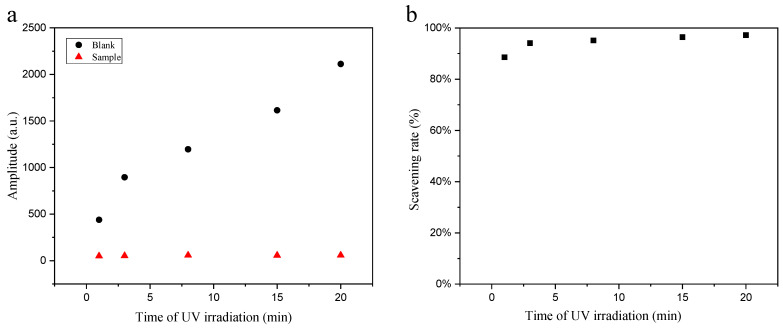
(**a**) Changes in EPR signal amplitude of DMPO-OH• adducts (generated with 5 mM H_2_O_2_ + 1 mM DMPO) as a function of irradiation time: Comparison between samples treated with 0.5 mg/mL SM extract and blank controls without SM Extract. (**b**) Dependence of the radical scavenging rate of SM extract on irradiation time.

The •OH radical SR of the samples under different UV irradiation durations is compared in Figure 5. Both the blank group (5 mM H_2_O_2_ + 1 mM DMPO) and the sample group (5 mM H_2_O_2_ + 1 mM DMPO + 0.5 mg/mL SM extract) were subjected to UV irradiated for varying durations, after which the EPR spectra of the DMPO-OH• adduct were recorded immediately. As presented in Figure 5a, the amplitude of the second characteristic peak in the EPR spectra demonstrated that the EPR signal amplitude of the DMPO-OH• adduct in the blank group exhibited a linear increase with prolonged irradiation time. In contrast, the corresponding signal amplitude in the sample group remained consistently low and showed negligible variation with increasing irradiation time, indicating that the introduced SM extract effectively scavenged the •OH radicals generated by the irradiation. Figure 5b depicts the •OH radical SR of the samples under different irradiation durations. Specifically, the SR of the sample reached 88.6% after 1 min of UV exposure, which increased to 95.1% at 8 min. With extended UV exposure time, the •OH radical SR continued to improve moderately, reaching 96.5% and 97.2% at 15 min and 20 min of irradiation, respectively. These results demonstrate the excellent •OH scavenging capacity of the SM extract. The high scavenging rate (nearly 90%) attained within the first minute of irradiation highlights its rapid-response antioxidant property, which is crucial for neutralizing burst production of •OH radicals in vivo—a process capable of inducing immediate cellular damage. Furthermore, the consistently high and gradually increasing scavenging rate observed over an extended irradiation period of up to 20 min reveals its enduring antioxidant resilience. The combination of rapid action and sustained stability is a hallmark of robust antioxidant systems and aligns with the proposed role of SM in protecting against chronic oxidative stress. Moreover, both overcomes described above are likely attributed to the contribution not only from silymarin flavonoids but also from essential trace elements.

The potent scavenging activities against both DPPH and •OH radicals, as demonstrated by EPR, provide robust evidence for the intrinsic chemical antioxidant capacity of the SM seed aqueous extract. However, it is crucial to critically consider the limitations when extrapolating these in vitro findings to potential biological effects. Factors such as bioavailability, cellular uptake, metabolic transformation, and the complex redox microenvironment in vivo may significantly modulate the antioxidant efficacy observed in simplified chemical systems. While DPPH offers a standardized measure and •OH represents a pathologically relevant oxidant, their reactions in a test tube do not fully replicate the spatial and temporal dynamics of radical generation and quenching in living organisms. Therefore, the results presented here establish a strong foundation for the extract’s antioxidant potential and justify its further investigation as a food–medicine resource. Nevertheless, future studies employing cellular models or in vivo assays are warranted to validate its physiological relevance and protective effects against oxidative stress.

## 3. Materials and Methods

### 3.1. Materials and Reagents

The materials including *Silybum marianum* seeds, refined extracts of silymarin flavonoids, standard of SBN containing SBNA and SBNB, and standard solutions of inorganic elements are itemized in detail in Supplementary Material Text S2. The CRM of tea (GBW07605) was purchased from Institute of Geophysical and Geochemical Prospecting for Certified Reference Materials (Langfang, China). Methanol (HPLC grade) was purchased from Merck KGaA (Darmstadt, Germany). Ethanol, glacial acetic acid, hydrogen peroxide (H_2_O_2_, 30%) and nitric acid (ultrapure grade) were obtained from Nanjing Chemical Reagent Factory (Nanjing, China). 1,1-Diphenyl-2-picrylhydrazyl radical (DPPH, ≥98.5%) was provided by Shanghai Macklin Biochemical Co., Ltd. (Shanghai, China), and 5,5-dimethyl-1-pyrroline N-oxide (DMPO) was by Dojindo China Co., Ltd. (Shanghai, China). All the reagents were of analytical grade unless stated otherwise and used without further purification. Wahaha purified water (Wahaha Group Co., Ltd., Hangzhou, China) was used for preparing mobile phases and solutions in the whole experiment process.

### 3.2. Preparation of Boiling Water Decoction and Extract of SM Seeds

SM decoction: 3.0 g of crushed SM seeds was weighed and placed in a 50 mL beaker, followed by the addition of 30 mL of boiling water (solid–liquid ratio 1:10, *w*/*v*). The mixture was heated to boiling in a water bath at 100 °C for 20 min, then transferred hot into a 50 mL centrifuge tube and centrifuged at 3000 r/min for 20 min. The supernatant was filtered through a 0.45 μm microporous cellulose membrane to obtain decoction of SM seeds for subsequent HPLC separation followed by ICP-MS analysis. Meanwhile, a blank decoction was prepared using the same volume of boiling water according to the identical procedure, except that SM seeds are not added.

SM extract: A total of 20 g of crushed SM seeds was weighed and immersed in 400 mL of deionized water (solid–liquid ratio 1:20, *w*/*v*). The mixture was heated at 100 °C for 20 min, after which the aqueous decoction was collected and filtered, and the filtrate was concentrated under reduced pressure using a rotary evaporator, yielding approximately 0.44 g of SM extract powder. The extract was frozen at −20 °C and stored until further use for preparing samples of varying concentrations for subsequent EPR analysis.

### 3.3. Apparatus

HPLC, ICP-MS and EPR instrumentations are itemized in detail in Supplementary Material Text S3.

### 3.4. Determination of Inorganic Elements in Fractionated Eluents of Boiling Water Decoction of SM Seeds (SM Decoction)

#### 3.4.1. RP-HPLC Separation of Silymarin Flavonoids

The decoction of SM seeds and blank decoction were subjected to RP-HPLC separation following our previous method [25] with minor modification as specified in Text S1. Two parallel replicates were prepared for both the sample and blank decoctions. One decoction solution was injected into the HPLC column four times, with an injection volume of 50 μL per run. The qualitative identification of silymarin flavonoids was carried out in comparison with the retention times of individual flavonoid references as well as standard SNB. To investigate the combination of flavonoids with elements, the total chromatographic runtime was divided into 9 consecutive, non-overlapping fractions based on the retention time of each flavonoid. Specifically, the initiation time of each subsequent peak was designated as the termination time of the preceding fraction, with detailed fractionation parameters listed in Table 2. The post-column eluents were collected in 50 mL beakers from the outlet of the PDA detector at predetermined time intervals, and the eluents corresponding to the same time interval were pooled in the same beaker.

#### 3.4.2. ICP-MS Analysis of Elements in the HPLC Fractionated Eluents

The collected HPLC eluents and blank eluents corresponding to different time intervals were evaporated to near dryness at low temperature. Three parallel replicates were taken for both the sample and blank eluents. After 5 mL HNO_3_ was placed, heated and evaporated to dryness, the digests were reconstituted with 2% (*v*/*v*) HNO_3_ solution, and made up to constant volumes of 3 mL. Thus, the digesting sample and the blank solutions for the determination of elements in the fractionated decoctions were prepared. The element concentrations in the HPLC effluents during each time interval were determined by ICP-MS under our previous conditions [34] with minor modification as described in Text S1.

During ICP-MS detection, analytical signals tend to drift over time; moreover, the presence of matrix effects can cause significant suppression of analyte signals, which compromises the accuracy and reproducibility of the results. To address these issues, the collected eluents of SM decoction from HPLC were evaporated to dryness at low temperature prior to analysis to remove abundant organic substances. This pretreatment procedure served two purposes: first, it harmonized the matrix of the test samples with that of the standard solutions, thereby mitigating matrix effects; second, it reduced polyatomic ion interferences, prevented sampler/skimmer cone clogging, and minimized memory effects. For mass spectral interferences induced by isobars, background noise, and the formation of oxides, hydroxides, or doubly charged ions, the interference was alleviated by selecting isotopes with high natural abundance and low interference potential. Herein, the isotopes ^75^As, ^114^Cd, ^59^Co, ^52^Cr, ^63^Cu, ^57^Fe, ^55^Mn, ^98^Mo, and ^64^Zn were chosen as the target isotopes for quantification. For interferences caused by adjacent-mass ions, correction equations were applied for elimination, exemplified as the following: ^75^As = ^75^As − 3.127 × ^77^ArCl, ^114^Cd = ^114^Cd − 0.02725 × ^118^Sn. Additionally, ^89^Y, an isotope with a moderate atomic mass, was employed as the internal standard during the experiments, which effectively counteracted the drift of analytical signals.

### 3.5. Measurement of Antioxidant Activity of Boiling Water Extract from SM Seeds (SM Extract)

EPR can directly and specifically detect paramagnetic substances, and it can avoid the common interference from inherent colored compounds in plant extracts compared to traditional spectrophotometry (such as UV–visible spectroscopy). In this study, two complementary radical systems were employed to evaluate the broad-spectrum antioxidant potential of SM extracts: (1) the stable nitrogen-centered DPPH radical, which serves as a standard probe for assessing hydrogen donor or electron transfer capacity; and (2) the highly reactive hydroxyl radical (•OH), generated in situ through the UV photolysis of H_2_O_2_. Due to its extremely short half-life, •OH is captured by DMPO to form a stable paramagnetic adduct, DMPO-OH•, for EPR detection. This combination of persistent radicals with transient and physiologically relevant radicals provides a more comprehensive evaluation of antioxidant activity.

All EPR measurements were conducted in accordance with our previous method [40] with minor modification as described in Text S1.

#### 3.5.1. Time-Dependence of DPPH Radical Scavenging Activity by SM Extracts

Using ethanol as the solvent, DPPH was subjected to serial dilution to prepare a standard solution with a concentration of 5 mM. Meanwhile, the SM extract was dissolved in deionized water to obtain a stock solution at a concentration of 5 mg/mL. Subsequently, 100 μL of the SM extract stock solution was mixed thoroughly with an equal volume (100 μL) of the 5 mM DPPH solution. The resulting mixture was then adjusted to a final volume of 1000 μL with ethanol, yielding a reaction system containing 0.5 mM DPPH and 0.5 mg/mL SM extract. EPR spectra of this reaction system were periodically recorded at different time intervals following the protocol described in Supplementary Text S1. The area under the characteristic DPPH peaks was calculated via double integration of the corrected EPR spectra using Bruker ESR Studio software (Version 1.90.0, Bruker BioSpin GmbH, Ettlingen, Germany). The DPPH radical scavenging rate (SR) was expressed via the formula (Equation (S1)) in Text S4.

#### 3.5.2. Dependence of DPPH Radical Scavenging Activity on SM Extract Concentration

Aliquots of 100 μL SM extract solutions with initial concentrations ranging from 1 to 8 mg/mL (or deionized water for the blank group) were mixed with equal volumes (100 μL) of 5 mM DPPH solution, and the mixture was diluted to a final volume of 1000 μL with ethanol. The reaction system was then incubated in the dark for 60 min, after which the mixture was analyzed via EPR spectroscopy.

#### 3.5.3. Time-Dependence of •OH Radical Scavenging Activity by SM Extract

The SM extract was dissolved in deionized water to prepare a stock solution with a concentration of 5 mg/mL. Equal volumes (90 μL each) of 10 mM DMPO, 50 mM H_2_O_2_ and 5 mg/mL SM extract solution (deionized water was used as the blank control) were mixed and diluted to a final volume of 900 μL with deionized water. Subsequently, 50 μL aliquots of the mixed solution were transferred into glass capillary tubes, which were then sealed with rubber putty. The sealed capillaries were irradiated using a xenon lamp positioned 35 cm away from the samples. The illumination was provided by a UV-Vis light source (model HSX-UV300, Beijing Newbit Technology Co., Ltd., Beijing, China) operating at a current of 15 A. The spectral output ranged from 200 nm to 2500 nm, encompassing the ultraviolet, visible, and near-infrared regions. After irradiation, the glass capillary tubes were placed into standard EPR quartz tubes for EPR spectroscopic analysis. EPR measurements were performed at irradiation time points ranging from 2 to 20 min to assess the time-dependent radical scavenging activity. The EPR signal intensity of DMPO-OH• adducts was quantified based on the amplitude value of the second characteristic peak in the spectra, defined as the difference between the peak height and trough depth. The •OH scavenging rate was calculated via the formula (Equation (S2)) in Text S4.

#### 3.5.4. Dependence of •OH Radical Scavenging Activity on SM Extract Concentration

Equal volumes (90 μL each) of 10 mM DMPO, 50 mM H_2_O_2_ and SM extract solutions at concentrations ranging from 1 to 8 mg/mL were mixed thoroughly. The resulting mixture was diluted to a final volume of 900 μL using deionized water. Subsequently, the mixture was then exposed to UV irradiation for 5 min, followed by EPR spectroscopic analysis to determine the •OH radical scavenging activity of the SM extract at different concentration gradients.

## 4. Conclusions

This study systematically evaluated the nutritional and functional benefits of tea-like infusions of SM seeds, with emphasis on trace element supplementation and antioxidant capacity, and established a reliable analytical protocol for correlating flavonoid components with inorganic elements in the herbal extracts. A combined HPLC and ICP-MS approach was employed for trace element profiling in boiling water decoction of SM seeds, where post-column effluents were fractionated according to the elution times of major silymarin components. Quantitative analysis revealed that the tea-like decoction is abundant in essential trace elements (Cu, Fe, Mn, Zn), with these elements primarily existing as ions or highly polar forms, while the levels of toxic elements (As, Cd, Co) were extremely low. Antioxidant activity assays via EPR spectroscopy demonstrated that the SM seed extract exhibits potent scavenging capacity against both DPPH and •OH radicals: at a concentration of 0.8 mg/mL, the extract achieved a SR exceeding 95% for DPPH radicals after 60 min of incubation, and an identical SR for •OH radicals generated by irradiated H_2_O_2_ solution. Collectively, these findings reveal that direct consumption of SM seed infusions can simultaneously enhance the body’s antioxidant defense system and supplement essential trace elements to maintain metabolic homeostasis, providing a scientific basis for its development as a natural antioxidant agent and potential application in clinical management of oxidative stress-related diseases. Furthermore, the integrated analytical method combining ICP-MS with EPR spectroscopy, proposed in this study, provides promising technical support for elucidating the pharmacological mechanisms of herbal medicines, optimizing their extraction with boiling water and expanding their applications in antioxidation research and clinical practice. Because RP-HPLC failed to detect the existence of weak interactions of silymarin flavonoids with trace elements due to the strong hydrophobic interaction between hydrophobic stationary phase and silymarin flavonoids, future studies employing complementary techniques, such as LC-MS and other spectroscopic analyses, would be useful to elucidate the coordination of trace elements and silymarin flavonoids in aqueous decoction. In addition, investigations into the bioavailability and physiological function of the binding states of SM flavonoids and trace elements would help to clarify the nutritional and pharmaceutical significance of the potential food–medicine dual-purpose resource.

## Figures and Tables

**Table 1:** Concentrations in collected solutions of different time range after HPLC separation (ng/mL).

Fraction Number	As	Cd	Co	Cr	Cu	Fe	Mn	Mo	Zn
First	0.03 (0.02)	0.10 (0.02)	0.23 (0.00)	2.71 (0.23)	46.3 (0.38)	16.1 (2.04)	6.19 (0.26)	1.49 (0.01)	27.3 (1.56)
Second	ND	ND	0.07 (0.00)	0.74 (0.03)	11.7 (0.66)	15.4 (2.25)	4.10 (0.53)	0.18 (0.00)	6.73 (0.92)
Third	0.04 (0.01)	ND	ND	ND	4.53 (0.64)	ND	2.01 (0.18)	0.09 (0.02)	ND
Fourth	ND	0.21 (0.01)	ND	2.54 (0.33)	6.03 (0.56)	15.8 (2.49)	3.92 (0.19)	ND	7.91 (1.06)
Fifth	ND	0.35 (0.01)	0.14 (0.01)	ND	1.78 (0.24)	ND	1.87 (0.05)	ND	5.05 (0.98)
Sixth	ND	0.06 (0.00)	0.07 (0.00)	0.08 (0.04)	3.40 (0.24)	ND	2.15 (0.16)	0.17 (0.01)	ND
Seventh	ND	0.08 (0.01)	ND	0.06 (0.01)	3.80 (0.11)	5.80 (2.30)	1.47 (0.08)	0.21 (0.01)	ND
Eighth	0.21 (0.00)	0.06 (0.00)	ND	ND	2.28 (0.06)	ND	1.82 (0.13)	ND	2.44 (0.17)
Ninth	ND	0.12 (0.00)	ND	0.22 (0.06)	2.07 (0.19)	10.5 (2.27)	1.80 (0.25)	ND	ND
Total concentration in digestion solution of HPLC effluent (ng/mL)	0.28	0.98	0.51	6.35	81.9	63.6	25.3	2.14	49.4
Total amount in 200 μL decoction (ng)	0.84	2.94	1.53	19.0	246	191	75.9	6.42	148
Total concentration in 30 mL decoction (ng/mL)	4.20	14.7	7.65	95.0	1230	955	380	32.1	740

ND represents “not detected”; data in parentheses are SD (n = 3).

**Table 2:** Time range of HPLC effluent collection.

Fraction Number	Compound	Time Range (min)	Fraction Number	Compound	Time Range (min)
First	—	0.0–2.7	Sixth	SDN	19.0–24.5
Second	—	2.7–4.7	Seventh	—	24.5–30.3
Third	—	4.7–7.0	Eighth	SBN	30.3–38.0
Fourth	TXF	7.0–15.0	Ninth	ISBN	38.0–45.0
Fifth	SCN	15.0–19.0			

## Data Availability

Data are contained within the article and Supplementary Materials.

## Supplementary Materials

The following supporting information can be downloaded at: https://www.mdpi.com/article/10.3390/molecules31061034/s1, Text S1: The separation condition of HPLC, determination condition of ICP-MS and measurement condition of EPR; Text S2: Samples and standard solutions; Text S3: HPLC, ICP-MS and EPR instrumentations; Text S4: The formulas used in EPR measurement; Figure S1: Preparative chromatogram of boiling water decoction from SM seeds (Peaks: 1. TXF; 2. SCN; 3. SDN; 4. SBNA; 5. SBNB; 6. ISBNA; 7. ISBNB).

## Abbreviations

The following abbreviations are used in this manuscript:

SM	*Silybum marianum*
TXF	Taxifolin
SCN	Silycristin
SDN	Silydianin
SBNA	Silybin A
SBNB	Silybin B
ISBNA	Isosilybin A
ISBNB	Isosilybin B
DPPH	2,2-Diphenyl-1-picrylhydrazyl
DMPO	5,5-Dimethyl-1-pyrroline N-oxide
TCM	Traditional Chinese medicine
RP-HPLC	Reversed-phase high-performance liquid chromatography
EPR	Electron paramagnetic resonance spectroscopy
ICP-MS	Inductively coupled plasma mass spectrometry (ICP-MS)
ICP-AES	Inductively coupled plasma atomic emission spectrometry
HPLC	High-performance liquid chromatography
LC-MS	Liquid chromatography–mass spectrometry
