# Supplementary material for: Insights into Antioxidant Activity and Trace Element Distribution of Aqueous Extract of Silybum marianum Seeds
Source: Molecules. 2026 Mar 19;31(6):1034. doi: 10.3390/molecules31061034 (PMC13029399; doi:10.3390/molecules31061034)
Supplement: Supplementary file 1 [file molecules-31-01034-s001.zip › molecules-4145718-supplementary.pdf]

Supplementary Materials

Insights into Antioxidant Activity and Trace Element Distribution of Aqueous Extract of Silybum Marianum Seeds

Li Quan [1,†], Yi-Xiao Wang [2,†], Xiu-Lan Cai [2], En-Chao Zhou [1,*], Xue-Wen Guo [2], Yi-Jun Chen [2] and Hong-Zhen Lian [2,*]

[1] Jiangsu University Key Laboratory of Tonifying Kidney and Anti-Senescence, The First Clinical Medical College, Nanjing University of Chinese Medicine, Nanjing 210023, China

[2] State Key Laboratory of Analytical Chemistry for Life Science, School of Chemistry & Center of Materials Analysis, Nanjing University, Nanjing 210023, China

* Correspondence: Correspondence: zhouenchao@njucm.edu.cn (E.-C.Z.); hzlian@nju.edu.cn (H.-Z.L.)

† These authors contributed equally to this work.

Text S1. The separation condition of HPLC, determination condition of ICP-MS and measurement condition of EPR.

HPLC separation was carried out on a 5 μm Kromasil C18 column with the dimension of 150 mm × 4.6 mm I.D. (Jiangsu Hanbang Science and Technology Co. Ltd, Huai'an, Jiangsu, China) at 30 °C. A trinary gradient elution employing methanol (A), water (B) and 1% aqueous acetic acid solution (C) was made at a flow rate of 1.0 mL/min. The linear gradient is: (0 min) 30% A-50% B-20% C, (5 min) 35% A-45% B-20% C, (20 min) 35% A-45% B-20% C, (25 min) 45% A-35% B-20% C, (60 min) 45% A-35% B-20% C. A sample volume of 50 μL was injected throughout the experiment. The silymarin compounds in effluent were monitored at 280 nm during the HPLC separation.

ICP-MS determination was carried under the operating conditions as follows: RF power: 1100 W; plasma gas flow rate: 16.0 L/min; auxiliary gas flow rate: 1.0 L/min; carrier gas flow rate: 0.95 L/min; sample lifting speed: 1.1 mL/min; ion lens voltage: 5.6 V; analog voltage: 1600 V; pulse voltage: 950 V; scan mode: peak-hopping; dwell time: 50 ms; scanning times: 3; integral time: 1000 ms.

EPR measurement was carried out in a glass capillary tube (inner diameter: 0.9-1.0 mm) containing approximately 50 μL of the sample solution sealed with rubber putty and placed into a standard EPR quartz tube (inner diameter: 3 mm). The tube was then placed in the resonator cavity of the EPR spectrometer. The settings used for EPR spectroscopy were as follows: central magnetic field, 338.40 mT; sweep width: 15 mT; microwave power: 10 mW; modulation frequency: 100 kHz; modulation amplitude: 0.2 mT; sweep time, 60 s; test temperature: 25 °C.

Text S2. Samples and standard solutions.

Silybum marianum seeds were provided by Jiangsu Jushoutang Biological Products Co., Ltd. (Taizhou, Jiangsu, China). Standard of SBN (a mixture of SBNA and SBNB, total SBN content ≥98%) was supplied by the National Institute for the Control of Pharmaceutical and Biological Products (Beijing, China). Refined extracts of TXF, SCN, SDN, SBNA, SBNB, ISBNA and ISBNB for retention time reference (the individual purities not certified) were provided by

the College of Traditional Chinese Pharmacy, China Pharmaceutical University (Nanjing, Jiangsu, China).

Standard solutions of inorganic elements As, Cd, Co, Cr, Cu, Fe, Mn, Mo and Zn with a concentration of 1.0 mg/mL were all prepared from spectroscopically pure metallic elements, oxides, or salts. Certified reference material (CRM) of tea (GBW07605) was obtained from the Institute of Geophysical and Geochemical Exploration, Ministry of Geology and Mineral Resources (Langfang, Hebei, China).

Text S3. HPLC, ICP-MS and EPR instrumentations.

The HPLC system used was Waters Alliance 2695 Separations Module equipped with a vacuum degasser, a quaternary pump, an auto-sampler, and a 996 UV-Vis photodiode-array detector (PDA) (Waters, Milford, MA, USA). The separation was controlled and the chromatograms were recorded by a Waters Empower chromatography manager system.

The ICP-MS used was Perkin-Elmer SCIEX ELAN 9000 Inductively Coupled Plasma Mass Spectrometer (Perkin-Elmer, Inc., Wellesley, MA, USA) equipped with a glass concentric nebulizer and a cyclonic spray chamber (Glass Expansion, Romainmotier, Switzerland).

The EPR used was Magnettech ESR5000 Benchtop EPR Spectrometer (Bruker BioSpin GmbH, Ettlingen, Germany).

Text S4. The formulas used in EPR measurement.

The DPPH radical scavenging rate (SR) is expressed by the following formula:

$SR= (A_0 - A) / A_0 \times 100\%$ ·· (S1)

where A_0 denotes the double integral area under the characteristic peaks of the EPR spectrum for the blank group, and A represents that of the EPR spectra for the sample groups.

The hydroxyl radical (•OH) scavenging rate (SR) is calculated according to the following formula:

$SR = (I_0 - I) / I_0 \times 100\%$ ··(S2)

where I_0 denotes the amplitude value of the second characteristic peak in the EPR spectrum for the blank group, and I represents the corresponding value for the sample groups.

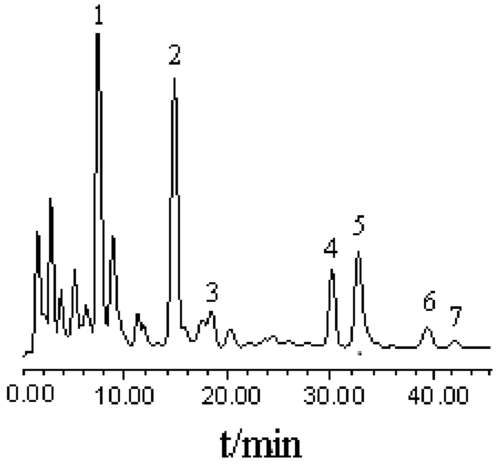

Figure S1. Preparative chromatogram of boiling water decoction from SM seeds (Peaks: 1. TXF; 2. SCN; 3. SDN; 4. SBNA; 5. SBNB; 6. ISBNA; 7. ISBNB).
